# Supplementary material for: NeuroCORD: A Language Model to Facilitate COVID-19-Associated Neurological Disorder Studies
Source: Int J Environ Res Public Health. 2022 Aug 12;19(16):9974. doi: 10.3390/ijerph19169974 (PMC9408703; doi:10.3390/ijerph19169974)
Supplement: Supplementary file 1 [file ijerph-19-09974-s001.zip › Figure S1.pdf]

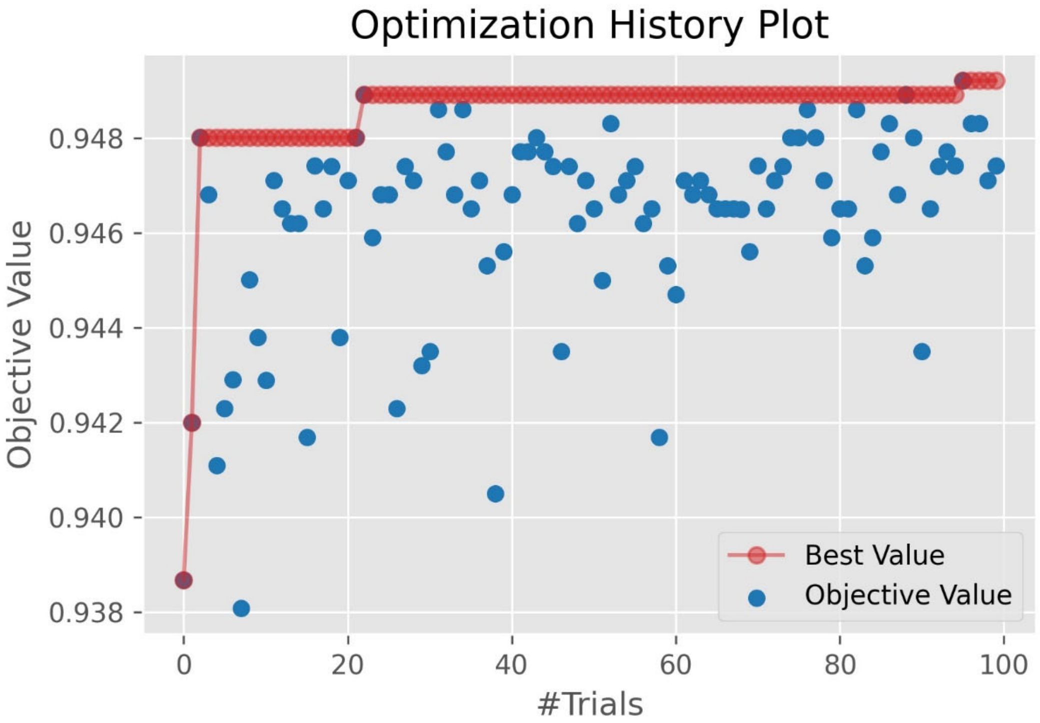

Figure S1. Parameter optimization result. X is the number of trails during the optimization process, Y represents the performance in accuracy. The individual dots represent the 10-fold cross scores of each combination tested, with the red line represented the overall best score.
