# Supplementary material for: NeuroCORD: A Language Model to Facilitate COVID-19-Associated Neurological Disorder Studies
Source: Int J Environ Res Public Health. 2022 Aug 12;19(16):9974. doi: 10.3390/ijerph19169974 (PMC9408703; doi:10.3390/ijerph19169974)
Supplement: Supplementary file 1 [file ijerph-19-09974-s001.zip › Supplementary tables.pdf]

Table S1. Relevant ratio based on keywords-based search.

TEXT LEVEL	RELEVANT	TOTAL	EST. RELEVANT RATIO
**TITLE**	447	529,397	0.1%
**ABSTRACTS**	1,691	385,194	0.5%
**FULL TEXTS**	3,427	186,530	2%

Table S2. Input processing approach optimization result.

**Datasets**	**Category**	**Abstract**	**Summary**	**Sentences**	**Sentence (Consensus)**
Training	Sample Size	2,561	2,561	20,244	
(Before 2020-12-31)	10-fold CV (ACC)	**0.972**	0.939	0.890	
Testing	Sample Size	819	819	7,328	819
(After 2020-12-31)	Testing (ACC)	**0.940**	0.902	0.819	0.921
